# Optimization of carbon source and glucose feeding strategy for improvement of L-isoleucine production by *Escherichia coli*

**DOI:** 10.1080/13102818.2015.1006899

**Published:** 2015-02-04

**Authors:** Jian Wang, Bing Wen, Qingyang Xu, Xixian Xie, Ning Chen

**Affiliations:** ^a^ Department of Bioengineering, College of Biological and Agricultural Engineering, Jilin University, Changchun, Jilin, China; ^b^ National and Local United Engineering Lab of Metabolic Control Fermentation Technology, Tianjin University of Science and Technology, Tianjin, China; Key Laboratory of Industrial Microbiology of Education Ministry, Tianjin University of Science and Technology, Tianjin, China; ^c^ Tianjin Research Institute of Industrial Microbiology, Tianjin, China

**Keywords:** L-isoleucine, *E. coli*, carbon source, feeding strategy, acetate

## Abstract

Fed-batch cultivations of L-isoleucine-producing *Escherichia coli* TRFP (SG^r^, *α*-ABA^r^, with a pTHR101 plasmid containing a thr operon and ilvA) were carried out on different carbon sources: glucose, sucrose, fructose, maltose and glycerol. The results indicated that sucrose was the best initial carbon source for L-isoleucine production and then sucrose concentration of 30 g·L^−1^ was determined in the production medium. The results of different carbon sources feeding showed that the glucose solution was the most suitable feeding media. The dissolved oxygen (DO) of L-isoleucine fermentation was maintained at 5%, 15% and 30% with DO-stat feeding, respectively. The results indicated that when the DO level was maintained at 30%, the highest biomass and L-isoleucine production were obtained. The accumulation of acetate was decreased and the production of L-isoleucine was increased markedly, when the glucose concentration was maintained at 0.15 g·L^−1^ by using glucose-stat feeding. Finally, the glucose concentration was maintained at 0.10 g·L^−1^ and the DO level was controlled at approximately 30% during the whole fermentation period, using the combined feeding strategy of glucose-stat feeding and DO feedback feeding. The acetate accumulation was decreased to 7.23 g·L^−1^, and biomass and production of L-isoleucine were increased to 46.8 and 11.95 g·L^−1^, respectively.

## Introduction

L-isoleucine is an essential branched-chain amino acid and is, therefore, widely used in food, animal feed and medicine. *Corynebacterium glutamicum* is the predominant micro-organism for the industrial production of L-isoleucine,[1] but the fermentation period is long and the fermentation process is difficult to control.[2] We previously constructed an L-isoleucine-producing recombinant *Escherichia coli* TRFP containing the plasmid pTHR101 (thr operon and *ilv*A) and with double resistance towards sulphaguanidine (SG) and *α*-aminobutyric acid (*α*-ABA).[2] However, the production of L-isoleucine by *E. coli* TRFP was low because of high concentrations of acetate and NH_4_^+^.[3]

*E. coli* generates acetate as an undesirable by-product that has several negative effects on cell growth and protein production.[4] The accumulation of acetate can be reduced by optimizing the fermentation process and by genetic modification of the cells.[5] Acetate formation is strongly affected by the composition of the culture medium and the accumulation of acetate can be reduced by replacing glucose with other carbon sources.[4,6,7] Acetate formation can also be prevented by forcing glucose-limited cells to grow below the threshold specific growth rate. In fed-batch fermentation processes, both concentration of glucose and specific growth rate can be maintained below a certain critical value by adjusting the feeding rate of glucose.[4] It is important to select an appropriate feeding method, according to different cultivation conditions.[8] When a combined feeding strategy of pseudo-exponential feeding and glucose-stat feeding was used in L-tryptophan production by *E. coli*, the specific growth rate and glucose concentration were controlled at appropriate levels, leading to decreased acetate accumulation and increased biomass and L-tryptophan production.[8]

In this study, the effects of initial carbon source and feeding carbon source on L-isoleucine fermentation were investigated and different feeding strategies were applied to L-isoleucine fed-batch fermentation in order to decrease the accumulation of acetate and increase the production of L-isoleucine.

## Materials and methods

### Micro-organism and media

*E* *. coli* TRFP (SG^r^, *α*-ABA^r^) carrying a pTHR101 plasmid containing a thr operon and ilvA was derived by repeated compound mutagenesis from *E. coli* K12. The L-isoleucine-producing *E. coli* TRFP, which was used in this study, is capable of using sucrose as the carbon source and was obtained previously [2] and stored at the Culture Collection of Tianjin University of Science and Technology.

The seed medium contained the following components: 30 g·L^−1^ of glucose, 10 g·L^−1^ of yeast extract, 20 mL·L^−1^ of corn steep liquor, 10 g·L^−1^ of (NH_4_)_2_SO_4_, 5 g·L^−1^ of MgSO_4_·7H_2_O, 1 g·L^−1^ of KH_2_PO_4_ and 50 mg·L^−1^ of chloramphenicol. The fermentation medium for producing L-isoleucine contained the following components: 20 mL·L^−1^ of corn steep liquor, 4 g·L^−1^ of (NH_4_)_2_SO_4_, 1 g·L^−1^ of MgSO_4_·7H_2_O, 2 g·L^−1^ of KH_2_PO_4_, 0.1 g·L^−1^ of FeSO_4_·7H_2_O, 10 mg·L^−1^ of biotin and 50 mg·L^−1^ of chloramphenicol. It was supplemented with 20 g·L^−1^ of carbon source (glucose, sucrose, fructose, maltose and glycerol) or different concentrations of sucrose (10, 30, 50 and 70 g·L^−1^), depending on the experiment. The pH of the both seed and fermentation media was adjusted to 7.2 with 4 mol·L^−1^ NaOH.

### Culture methods

#### Fermentation in baffled flasks

A single colony of *E. coli* TRFP was inoculated into a 500 mL baffled flask containing 30 mL of seed medium and cultivated at 37° C with shaking at 200 r·min^−1^ for 12 h. Three millilitres of this culture were inoculated into a 500 mL baffled flask containing 30 mL of production medium and were cultivated at 37° C, with shaking at 200 r·min^−1^ for 36 h. In the cultivation process, 800 g·L^−1^ of carbon source (glucose, sucrose, fructose, maltose and glycerol) were fed into the baffled flask every two hours to maintain the concentration of sugar at 2–5 g·L^−1^, depending on the experiment.

#### Fermentation in a bioreactor

A 30 mL of the inoculum grown in the baffled flask was added aseptically to a 5 L seed fermenter (Biotech-2002 Bioprocess controller, Baoxing, Shanghai, China) containing 3 L of seed medium and cultivated at 37° C for 16 h. The culture grown in the seed fermenter was inoculated aseptically (10%, (v/v)) into 18 L of production medium in a 30 L fermenter. The temperature and dissolved oxygen (DO) level were maintained at 37 °C and 20%. The pH was adjusted to 7.2 (0–4 h), 7.0 (4–14 h) and 6.7 (14–36 h).[3] Once the initial 30 g·L^−1^ of sucrose was decreased to 2 g·L^−1^, 800 g·L^−1^ of glucose or sucrose solution was continuously fed into the fermenter to maintain the sugar concentration at 2 g·L^−1^, for the comparison of different feeding carbon sources. The DO-stat, glucose-stat strategy and combined feeding strategy of glucose-stat feeding and DO feedback feeding were adopted to control the timing of adding feed. Details of the DO-stat strategy, glucose-stat strategy and combined feeding strategy of glucose-stat feeding and DO feedback feeding operation have been described elsewhere.[8]

### Analysis of fermentation products

The DO, pH and temperature were measured automatically with electrodes attached to the fermenters. The optical density (OD) and dry cell weight (DCW) were determined according to the method of Wang et al. [5]. The concentration of glucose was monitored by an SBA-40C biosensor analyser (Biology Institute of Shandong Academy of Sciences, China). The concentration of acetate was measured with a Bioprofile 300A biochemical analyser (Nova Biomedical, Waltham, MA, USA). The concentration of L-isoleucine in the broth was derivatized with an equal amount of 1% (m/m) fluoro-2,4-dinitrobenzene (FDNB) dissolved in acetonitrile and determined by high-performance liquid chromatography (HPLC) using an Agilent 1200 instrument (Agilent Technologies, Santa Clara, CA, USA) equipped with an Agilent C18 (150 mm × 4.6 mm, 3.5 µm) column. A 1 mL·min^−1^ mobile phase using a solvent of acetonitrile/NaAc was applied to the column. The column was operated at 33° C. The detection wavelength was 360 nm. The concentration of carbon source in the broth was determined using HPLC.[9]

## Results and discussion

### Comparison of different carbon sources in L-isoleucine fermentation

**Figure 1.**
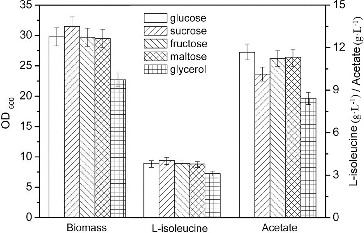
Effects of carbon source on L-isoleucine fed-batch fermentation by *E. coli* TRFP in baffled flasks.

Glucose, sucrose, fructose, maltose and glycerol are carbon sources used for growth and amino acid production of *E. coli*. However, the effects of these carbon sources on L-isoleucine production with *E. coli* TRFP have not been tested so far, to the best of our knowledge. Therefore, we compared the L-isoleucine production by *E. coli* TRFP in the presence of glucose, sucrose, fructose, maltose and glycerol. In L-isoleucine fermentation, the highest concentration of acetate (11.67 g·L^−1^) was accumulated with *E. coli* TRFP grown on glucose and the lowest accumulation of acetate (8.43 g·L^−1^) was obtained on glycerol. However, both biomass (22.7 g·L^−1^) and L-isoleucine production (3.12 g·L^−1^) on glycerol were lower than those on glucose, which may be due to the low utilization capacity of the L-isoleucine-producing strain for glycerol (Figure 1). Glucose is the most widely utilized raw material in industrial fermentations with *E. coli*, because it is relatively inexpensive and is a preferred carbon source.[10] Growth of *E. coli* on excess glucose under aerobic conditions causes the formation of acidic by-products, the most common of which is acetate.[8,11,12] Glycerol is an energy-poor carbon source, which has enhanced its biotechnology importance as a carbon source.[13] When *E. coli* grows aerobically on glycerol, this carbon source is incorporated into the central metabolism as dihydroxyacetone phosphate (DHAP) and DHAP can participate in both gluconeogenic and glycolytic processes.[14] The specific growth rate decreases and low level or no acetate production is detected when *E. coli* is cultured on glycerol.[15]

When sucrose was used as a carbon source, the highest biomass (31.5 g·L^−1^) and L-isoleucine production (4.05 g·L^−1^) were obtained, which were 5.71% and 2.79% higher when compared with glucose as a carbon source. Moreover, the accumulation of acetate on sucrose was lower than that on glucose. Thus, sucrose was the best carbon source for L-isoleucine fermentation, which was consistent with the result of L-threonine fermentation by *E. coli* TRFC.[16]

### Effect of initial sucrose concentration on L-isoleucine fermentation

**Figure 2.**
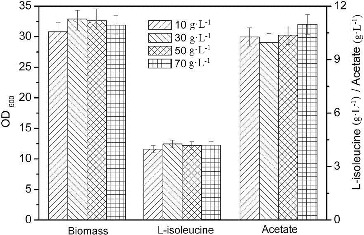
Effect of different initial sucrose concentrations on L-isoleucine fed-batch fermentation by *E. coli* TRFP in baffled flasks.

The L-isoleucine fermentation was carried out in baffled flasks. The growth and biomass of *E. coli* were affected by the carbon source concentration. The *E. coli* strain grew faster at low initial sucrose concentration, but higher biomass was achieved at high initial concentration of sucrose.[16] Specific growth rate is an important parameter in the fermentation process due to its impact on plasmid stability and the formation of acetate.[17] Acetate concentration and its production rate were increased with higher growth rate.[8] The carbon source concentration also changes the carbon/nitrogen (C/N) ratio, which affects the metabolism of *E. coli*.[18] The specific acetate, pyruvate and CO_2_ production rates tended to increase as the C/N ratio increased [18] and high concentration of pyruvate can increase the formation of L-isoleucine and acetate. Improved tricarboxylic acid (TCA) cycle capacity led to low excretion of acetate.[19] As shown in Figure 2, the highest biomass (32.7 g·L^−1^) and production of L-isoleucine (4.27 g·L^−1^) and the lowest accumulation of acetate were obtained with initial sucrose concentration of 30 g·L^−1^. When the initial sucrose concentration was above 30 g·L^−1^, both biomass and L-isoleucine production were decreased with the increase of initial sucrose concentration, while the excretion of acetate was increased. Thus, 30 g·L^−1^ sucrose was added in the production medium of L-isoleucine.

### Comparison of different feeding carbon sources on L-isoleucine fermentation

**Figure 3.**
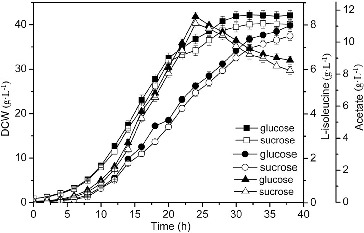
Effect of different feeding carbon sources on L-isoleucine fed-batch fermentation by *E. coli* TRFP.

Note: DCW (squares), production (circles) of L-isoleucine, concentration of acetate (upright triangles).

Substrate feeding is important for the production of L-isoleucine. The results indicated that higher biomass (42.2 g·L^−1^) and production of L-isoleucine (7.98 g·L^−1^) were obtained with glucose feeding, which were increased by 4.21% and 6.11% as compared to those with sucrose feeding. It may be because of the rapid utilization of glucose (Figure 3).[14] The glucose solution was selected as a feeding carbon source, due to the high biomass and L-isoleucine production.

### Effect of feeding strategy on L-isoleucine fermentation

Many fed-batch strategies have been developed to achieve high cell density cultivation of *E. coli* with the specific purpose of preventing the accumulation of acetate.[20,21] The accumulation of acetate was decreased by maintaining the glucose concentration at a low level. According to different cultivation conditions, selecting an appropriate feeding method was important for the formation of the desired product.

### L-isoleucine fermentation with DO-stat feeding

**Figure 4.**
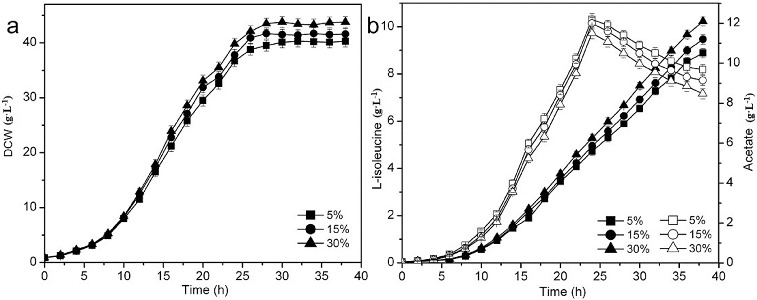
Effect of different DO concentrations on L-isoleucine fed-batch fermentation by *E. coli* TRFP with DO-stat feeding. Biomass (a); L-isoleucine (filled symbols) and acetate (open symbols) production (b).

The accumulation of acetate in the medium is a common problem of high cell concentration cultures. An imbalance between the glycolytic pathway and the tricarboxylic acid (TCA) cycle caused by the oxygen-limited culture conditions or a high concentration of glucose in the medium during aerobic growth results in the excretion of acetate.[22] Both the TCA cycle and acetate excretion can be affected by the transition from unlimited to limited oxygen supply, thereby altering cellular metabolism and protein production capability.[23] In this study, the DO levels were controlled at 5%, 15% and 30%, with DO-stat feeding. As shown in Figure 4, when the DO level was maintained at 30%, the lowest concentration of acetate was accumulated and highest concentration of acetate (3.03 g·L^−1^) was reused, obtaining the highest biomass (43.8 g·L^−1^) and L-isoleucine production (10.2 g·L^−1^). At low oxygen, both genes of the Pta-AckA pathway and the gene of PoxB seem to be transcribed, while at high oxygen only the Pta-AckA pathway genes are transcribed. The enhanced acetate accumulation at low DO was most probably the result of low TCA cycle activity and altered transcription levels of genes associated with glucose and acetate metabolism.[23] The transcription of the gluconeogenesis (*pckA*, *ppsA*) and the anaplerotic pathway (*ppc*, *sfcA*) genes was lower at low DO level than that at high DO level, which contributes to the accumulation of pyruvate and acetyl-CoA causing acetate accumulation. Under the conditions of limited oxygen, the low transcription levels of acetyl-CoA synthetase and isocitrate lyase are an indication of low acetate uptake and glyoxylate shunt activity, respectively.[24] Acetate of 2.86 g·L^−1^ was reused at DO level of 15% and 2.52 g·L^−1^ at DO level of 5%. Thus, the relatively high DO maintained in L-isoleucine fermentation was beneficial for L-isoleucine formation.

### L-isoleucine fermentation with glucose-stat feeding

**Figure 5.**
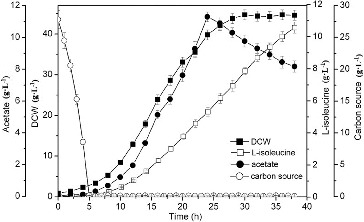
L-isoleucine fed-batch fermentation by *E. coli* TRFP with glucose-stat feeding.

In fermentation processes, glucose concentration is an immediate and effective control measure for microbial metabolism. The concentration of glucose could be maintained at a low level without starving the cells by using a glucose predictive and feedback computer-controlled fermentation system.[25] When the initial sucrose was depleted, the glucose solution was fed into the fermenter to maintain the concentration of residual glucose at 0.15 g·L^−1^. As shown in Figure 5, the excretion of acetate was decreased to 8.17 g·L^−1^ and the biomass and production of L-isoleucine were increased to 44.7 and 10.57 g·L^−1^, respectively.

### L-isoleucine fermentation with a combined feeding strategy of glucose-stat feeding and DO feedback feeding

Several parameters can reflect the control of a carbon source in fermentation processes such as pH, DO and concentration of carbon dioxide in the off-gas. One useful approach involves applying glucose pulses to the feed and observing the response of DO to those pulses.[4,26–28] DO-stat feeding can be used to avoid substrate overfeeding and O_2_ limitation. When the equipment meets the oxygen demand of cells, the glucose-stat feeding was used and the DO feedback feeding was used when the equipment cannot meet the oxygen demand of cells. The combined feeding strategy of glucose-stat feeding and DO feedback feeding was applied in L-isoleucine fermentation.

**Figure 6.**
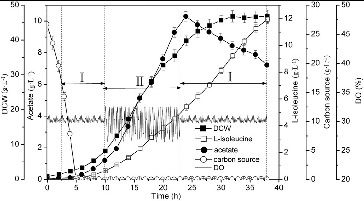
L-isoleucine fed-batch fermentation by *E. coli* TRFP with a combined feeding strategy of glucose-stat feeding and DO feedback feeding. Glucose-stat feeding (I); DO feedback feeding (II).

The excretion of acetate was decreased with the concentration of glucose controlled below the threshold for ‘Crabtree’ effect occurrence. When the glucose-stat feeding was used in L-isoleucine fermentation, the glucose concentration was controlled at a low level, leading to a decrease of acetate accumulation and an improvement of biomass and L-isoleucine production. The online detection of overflow metabolism can be enabled using a standard DO probe and a simple feedback algorithm could then be used to adjust the glucose feed rate to avoid overflow metabolism.[29] Online detection of acetate formation makes it possible to avoid overflow metabolism using feedback control of the glucose feed rate. A balance between oxygen transfer and oxygen uptake can be achieved with the strategies manipulating the feed rate to maintain a constant DO concentration to preclude anaerobic conditions.[30] As shown in Figure 6, the DO was maintained at approximately 30% by adjusting the agitation and aeration rates and using the DO feedback feeding and the glucose concentration was maintained at approximately 0.10 g·L^−1^. As compared with using only glucose-stat feeding, the biomass (46.8 g·L^−1^) and production of L-isoleucine (11.95 g·L^−1^) were increased by 4.70% and 13.06%, respectively, and the accumulation of acetate (7.23 g·L^−1^) was decreased by 11.51% by using this combined feeding strategy.

As compared to our previous work,[3] the accumulation of acetate was decreased by 36.02% and the biomass and production of L-isoleucine were increased by 17.59% and 68.31%, respectively, with the optimization of carbon source and glucose feeding strategy in this study. However, the accumulation of acetate was high. It has been reported that cell growth is inhibited by acetate concentration above 1.5 g·L^−1^.[31] The amount of accumulated acetate was reduced significantly by the elimination of Pta and AckA and increasing the anaplerotic flux or utilizing mutants with altered glucose transport.[32] The L-isoleucine production obtained in *E. coli* TRFP was relatively low. The production of L-isoleucine can be increased by overexpression of key genes for L-isoleucine biosynthesis and deletion of some important genes repressing L-isoleucine production and modification of transport proteins of L-isoleucine. [1,33]

## Conclusions

This study demonstrated that the use of 30 g·L^−1^ sucrose as an initial carbon source and glucose as feeding media increased the L-isoleucine production by *E*. *coli* TRFP substantially. By employing the combined feeding strategy of glucose-stat feeding and DO feedback feeding, the accumulation of acetate in the broth was decreased to 7.23 g·L^−1^, and as a result the biomass and production of L-isoleucine were increased to 46.8 and 11.95 g·L^−1^, respectively. To additionally improve L-isoleucine production by *E*. *coli* TRFP, further studies are needed by optimization of the fermentation process and construction of new strains.

## Disclosure statement

No potential conflict of interest was reported by the authors.
